# Hyperbaric Oxygen Treatment Following Mid-Cervical Spinal Cord Injury Preserves Diaphragm Muscle Function

**DOI:** 10.3390/ijms21197219

**Published:** 2020-09-30

**Authors:** Ashley J. Smuder, Sara M. Turner, Cassandra M. Schuster, Aaron B. Morton, J. Matthew Hinkley, David D. Fuller

**Affiliations:** 1 Department of Applied Physiology and Kinesiology, University of Florida, Gainesville, FL 32611, USA; mortona@health.missouri.edu (A.B.M.); james.kinkley@adventhealth.com (J.M.H.); 2 Breathing Research and Therapeutics, University of Florida, Gainesville, FL 32610, USA; dfuller@phhp.ufl.edu; 3 Department of Physical Therapy, University of Florida, Gainesville, FL 32610, USA; search.create.write@gmail.com (S.M.T.); c.schuster@ufl.edu (C.M.S.); 4 McKnight Brain Institute, University of Florida, Gainesville, FL 32610, USA

**Keywords:** respiratory, atrophy, oxidative stress, antioxidant, reactive oxygen species

## Abstract

Oxidative damage to the diaphragm as a result of cervical spinal cord injury (SCI) promotes muscle atrophy and weakness. Respiratory insufficiency is the leading cause of morbidity and mortality in cervical spinal cord injury (SCI) patients, emphasizing the need for strategies to maintain diaphragm function. Hyperbaric oxygen (HBO) increases the amount of oxygen dissolved into the blood, elevating the delivery of oxygen to skeletal muscle and reactive oxygen species (ROS) generation. It is proposed that enhanced ROS production due to HBO treatment stimulates adaptations to diaphragm oxidative capacity, resulting in overall reductions in oxidative stress and inflammation. Therefore, we tested the hypothesis that exposure to HBO therapy acutely following SCI would reduce oxidative damage to the diaphragm muscle, preserving muscle fiber size and contractility. Our results demonstrated that lateral contusion injury at C3/4 results in a significant reduction in diaphragm muscle-specific force production and fiber cross-sectional area, which was associated with augmented mitochondrial hydrogen peroxide emission and a reduced mitochondrial respiratory control ratio. In contrast, rats that underwent SCI followed by HBO exposure consisting of 1 h of 100% oxygen at 3 atmospheres absolute (ATA) delivered for 10 consecutive days demonstrated an improvement in diaphragm-specific force production, and an attenuation of fiber atrophy, mitochondrial dysfunction and ROS production. These beneficial adaptations in the diaphragm were related to HBO-induced increases in antioxidant capacity and a reduction in atrogene expression. These findings suggest that HBO therapy may be an effective adjunctive therapy to promote respiratory health following cervical SCI.

## 1. Introduction

Cervical spinal cord injury (SCI) compromises respiratory function as a result of damage to respiratory neural circuitry required for diaphragm muscle contraction [1]. The diaphragm is the primary muscle of inspiration, and maintenance of proper inspiratory control is critical for ventilation [2]. Following cervical SCI, the development of diaphragm dysfunction increases the risk of respiratory complications, morbidity and mortality as a result of atelectasis, pneumonia and ventilator dependence [3]. Therefore, preserving diaphragm muscle health by mitigating atrophy and improving contractility should facilitate respiratory rehabilitation and positive respiratory outcomes after cervical SCI.

Disruption of redox balance in favor of an oxidative environment occurs after SCI [4] and appears to be a fundamental component of SCI-induced diaphragm injury [5]. Studies of mechanical ventilation show that supraphysiological reactive oxygen species (ROS) production in the inactive diaphragm acts as an upstream trigger to enhance proteolytic breakdown of diaphragm muscle [6]. Furthermore, in the acute phase following cervical SCI, the rat diaphragm muscle exhibits significant muscle atrophy and contractile dysfunction that is associated with lipid peroxidation and an increased rate of mitochondrial hydrogen peroxide emission [5]. Administration of the antioxidant Trolox immediately following SCI mitigates much of the diaphragm weakness while also reducing mitochondrial and oxidative damage [5]. These findings suggest that interventions intended to prevent aberrant ROS production and oxidative modification of muscle contractile proteins may attenuate respiratory impairment after cervical SCI.

Hyperbaric oxygen (HBO) therapy utilizes the delivery of 100% oxygen delivered above one atmosphere absolute (ATA). HBO is routinely used in clinical practice for treatment of ischemic diseases, to promote wound healing [7] and for decompression sickness [8]. There is also growing preclinical evidence indicating therapeutic potential for HBO treatment to enhance recovery of skeletal muscle health after injury [9,10,11]. Rodent studies confirm that both acute and chronic exposure to HBO can provoke substantial increases in skeletal muscle antioxidant enzyme expression [12]. Given that cervical SCI increases oxidative stress in the diaphragm and antioxidant therapy can mitigate diaphragm atrophy [5], we reasoned that HBO therapy could promote diaphragm muscle health following SCI. Accordingly, we tested the hypothesis that HBO delivered in the acute phase after cervical SCI can preserve diaphragm redox balance, thereby leading to reduced diaphragm atrophy and preserved contractility.

## 2. Results

### 2.1. Biological Response to Experimental Treatments

No differences in body weight existed between groups prior to the initiation of the experimental protocol non-injured, room air exposure (CON) = 240.1 ± 2.7 g; lateral-cervical spinal cord contusion (SCI) = 230.8 ± 4.3 g; lateral-cervical spinal cord contusion, HBO therapy (SCI + HBOT) = 232.0 ± 2.9 g). Upon completion of the experimental treatments, CON animals remained weight stable, SCI animals lost an average of 10.7% of their initial body weight and SCI + HBOT animals lost 8.7% of their initial body weight. Final body weight of SCI and SCI + HBOT animals was significantly reduced compared to CON animals.

### 2.2. HBO Treatment after SCI Preserves Specific Force Production and Cross-Sectional Area

**Figure 1:**
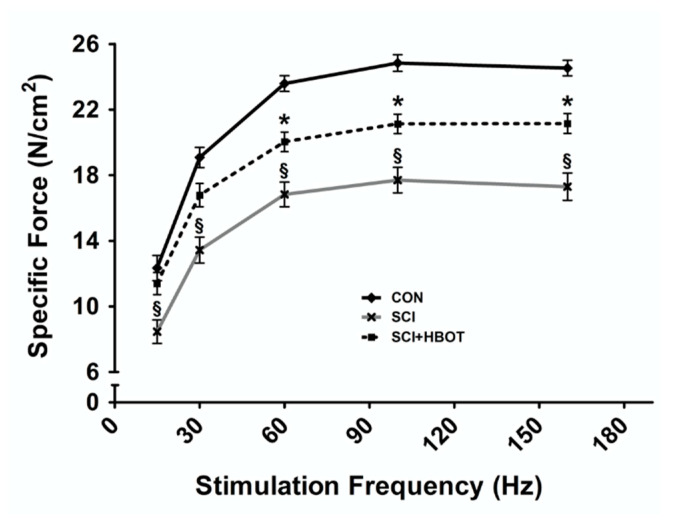
Diaphragm muscle force–frequency response. Values are mean ± SEM. § Significantly different versus all groups (*p* < 0.05). * Significantly different versus CON group (*p* < 0.05). CON—non-injured, room air exposure; SCI—lateral-cervical spinal cord contusion, room air exposure; and SCI + HBOT—lateral-cervical spinal cord contusion, HBO therapy.

The diaphragm muscle force–frequency response was significantly diminished in SCI rats that experienced lateral-cervical contusion injury compared to CON rats at all stimulation frequencies tested (Figure 1). Ten days of HBO treatment initiated one day following SCI was sufficient to preserve diaphragm muscle force production at submaximal stimulation frequencies of 15–30 Hz. At stimulation frequencies of 60–160 Hz, diaphragm muscle force production from the SCI + HBOT group remained significantly elevated compared to the SCI group, but specific force was depressed compared to the CON group.

**Figure 2:**
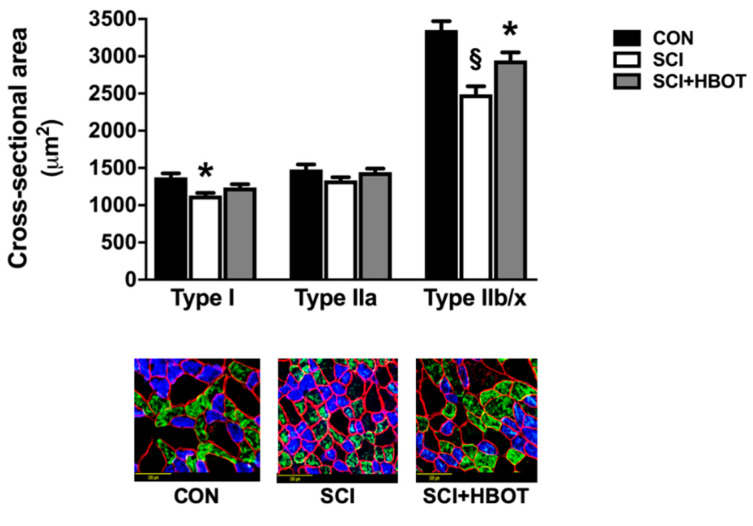
Diaphragm muscle cross-sectional area and fiber typing. Representative fluorescent staining of myosin heavy chain (MHC) I (type I) (DAPI filter/blue), MHC IIa (type IIa) (FITC filter/green), MHC IIb/IIx (type IIb/x) (non-stained) and dystrophin (Rhodamine filter/red) proteins in diaphragm samples are shown below the graph. Scale bar = 100 μm. Values are mean ± SEM. § Significantly different versus all groups (*p* < 0.05). * Significantly different versus CON group (*p* < 0.05). CON—non-injured, room air exposure SCI—lateral-cervical spinal cord contusion, room air exposure; and SCI + HBOT—lateral-cervical spinal cord contusion, HBO therapy.

Lateral-cervical contusion at C3/4 resulted in significant atrophy of type I and type IIb/x diaphragm muscle fibers compared to fiber cross-sectional area (CSA) from non-injured rats, with no difference in type IIa CSA observed between groups (Figure 2). Diaphragm muscle fiber CSA of type I fibers in the SCI + HBOT group was not significantly different between either CON or SCI rats. However, type IIb/x fiber CSA was significantly greater in the SCI + HBOT rats compared to SCI, but was also significantly reduced compared to CON rats.

### 2.3. Mitochondrial Function Is Maintained Following SCI and HBO Treatment

**Figure 3:**
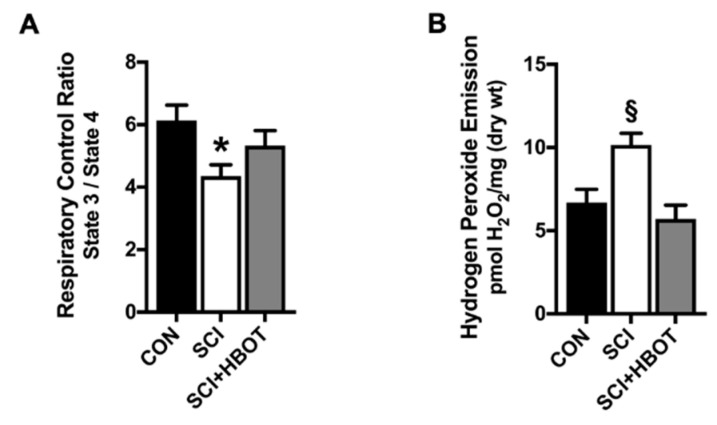
(**A**) Mitochondrial respiration and (**B**) mitochondrial reactive oxygen species (hydrogen peroxide) emission. Values are mean ± SEM. § Significantly different versus all groups (*p* < 0.05). * Significantly different versus CON group (*p* < 0.05). CON—non-injured, room air exposure; SCI—lateral-cervical spinal cord contusion, room air exposure; and SCI + HBOT—lateral-cervical spinal cord contusion, HBO therapy.

Diaphragm muscle mitochondrial function and ROS production were measured using permeabilized muscle fibers. Assessment of mitochondrial oxygen consumption in the diaphragm of SCI rats showed a significant reduction in respiratory control ratio (RCR) compared to CON rats (Figure 3A). In addition, hydrogen peroxide emission was significantly elevated in the diaphragm of SCI rats compared to both CON and SCI + HBOT rats (Figure 3B). No significant difference existed in state 3 respiration or state 4 respiration between groups (data not shown).

### 2.4. HBO Therapy Upregulates Endogenous Antioxidant Expression in the Diaphragm

**Figure 4:**
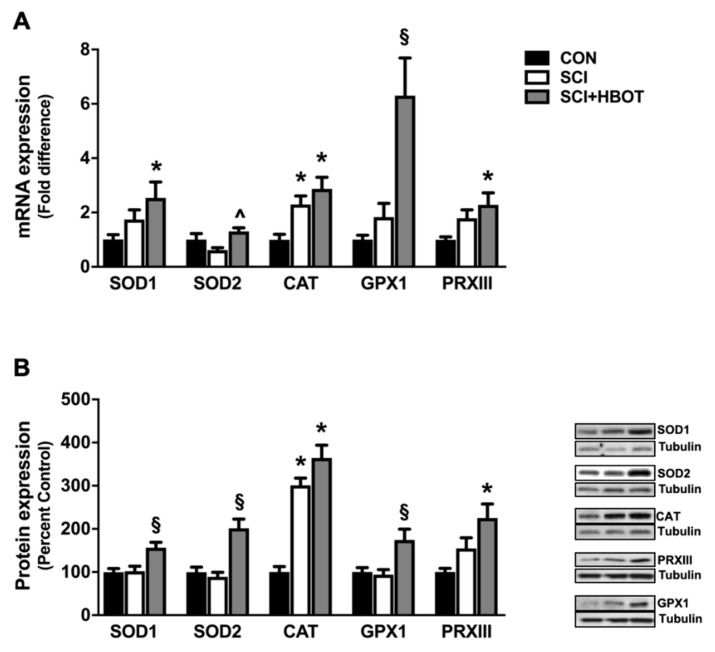
(**A**) Diaphragm mRNA expression of superoxide dismutase 1 (SOD1), superoxide dismutase 2 (SOD2), catalase (CAT), glutathione peroxidase 1 (GPX1) and peroxiredoxin III (PRXIII). (**B**) Diaphragm protein expression of SOD1, SOD2, CAT, GPX1 and PRXIII. Representative Western blot images are shown to the right of the graph. Values are mean ± SEM. § Significantly different versus all groups (*p* < 0.05). * Significantly different versus CON group (*p* < 0.05). ^ Significantly different versus SCI group (*p* < 0.05). CON—non-injured, room air exposure; SCI—lateral-cervical spinal cord contusion, room air exposure; and SCI + HBOT—lateral-cervical spinal cord contusion, HBO therapy.

HBO therapy induced a significant upregulation of endogenous antioxidant enzyme expression of superoxide dismutase 1 (SOD1), superoxide dismutase 2 (SOD2), catalase, glutathione peroxidase I (GPXI) and peroxiredoxin III (PRXIII) (Figure 4A,B). Specifically, diaphragm mRNA expressions of SOD1 and PRXIII were significantly increased in the SCI + HBOT rats compared to CON rats, and GPXI expression was increased compared to both CON and SCI rats. Gene expression of SOD2 was also significantly elevated in the SCI + HBOT rats compared to SCI rats. Catalase gene expression was significantly elevated in both SCI and SCI + HBOT rats compared to CON rats. The changes in mRNA expression were translated to the level of the protein, as catalase protein content was also significantly elevated in the SCI and SCI + HBOT groups compared to the CON group. In addition, PRXIII protein content was increased in the diaphragm of SCI + HBOT rats compared to CON rats, and SOD1, SOD2 and GPXI were all increased in SCI + HBOT rats compared to CON and SCI rats.

### 2.5. SCI Enhances Transcription of Inflammatory and Atrophy Markers in the Diaphragm

**Figure 5:**
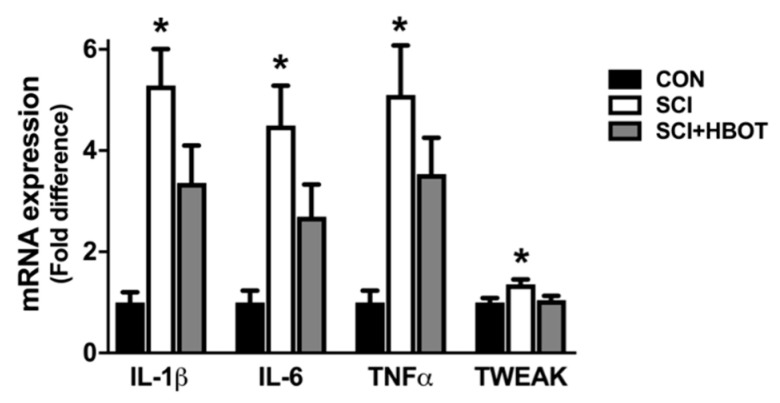
mRNA expression of interleukin 1β (IL-1β), IL-6, tumor necrosis factor α (TNFα) and TNF-related weak inducer of apoptosis (TWEAK). Values are mean ± SEM. * Significantly different versus CON group (*p* < 0.05). CON—non-injured, room air exposure; SCI—lateral-cervical spinal cord contusion, room air exposure; and SCI + HBOT—lateral-cervical spinal cord contusion, HBO therapy.

**Figure 6:**
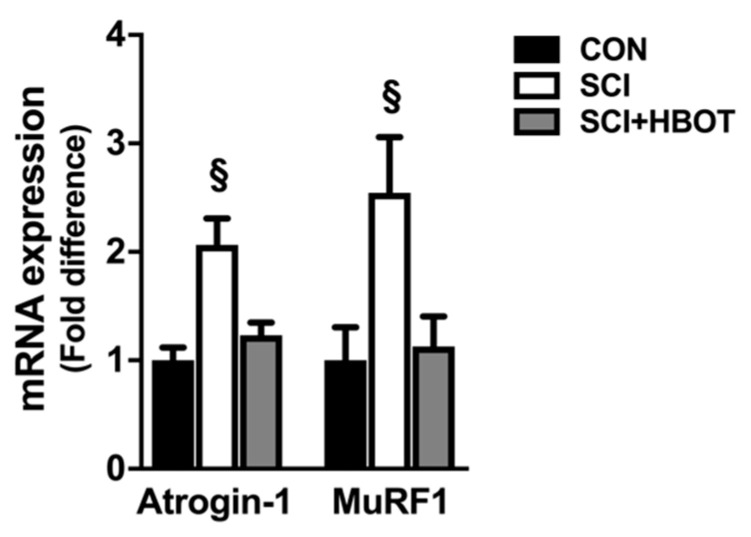
mRNA expression of Atrogin-1/MaFbx and MuRF1. Values are mean ± SEM. § Significantly different versus all groups (*p* < 0.05). CON—non-injured, room air exposure; SCI—lateral-cervical spinal cord contusion, room air exposure; and SCI + HBOT—lateral-cervical spinal cord contusion, HBO therapy.

Gene expression of the cytokines interleukin-1β (IL-1β), IL-6, tumor necrosis factor α (TNFα) and TNF-related weak inducer of apoptosis (TWEAK) were elevated in the diaphragm of SCI rats compared to CON rats (Figure 5). HBO therapy had no significant effect on the mRNA expression of these transcripts in the diaphragm, as no differences existed between the SCI + HBOT group compared to any other experimental group. In addition, diaphragm muscle mRNA expression of the E3 ligases Atrogin-1/MaFbx and MuRF1 were assessed as markers of enhanced protein degradation. Both transcripts were significantly elevated in the SCI group following lateral-cervical spinal contusion injury compared to the CON and SCI + HBOT groups (Figure 6).

## 3. Discussion

The diaphragm is particularly sensitive to the progression of disease and demonstrates rapid plasticity following inactivity [13,14,15,16]. Disruption of the phrenic motoneuron pool as a result of SCI leads to a reduction in diaphragm contraction and loading, which triggers remodeling [17,18]. The diaphragm response to cervical SCI involves temporal changes in myogenic signaling and activation of an acute atrophic response that is associated with increased inflammatory markers and pathological ROS production [5,13]. Previous work has shown that antioxidant therapies can mitigate the negative impact of SCI on diaphragm function independent of neurological considerations [5]. Our findings provide additional evidence that maintaining diaphragm muscle redox balance following cervical contusion injury effectively ameliorates diaphragm dysfunction as daily HBO therapy enhanced oxidative capacity, reduced ROS accumulation and preserved diaphragm muscle fiber size and contractility.

### 3.1. HBO Therapy Attenuates Diaphragm Atrophy and Contractile Dysfunction after SCI

Cervical SCI can result in rapid diaphragm wasting [5,13,17,19,20]. The diaphragm has been histologically, molecularly and functionally characterized in preclinical models including hemilesion of the C2 spinal cord and contusion at C3 and C4 [5,13,17,19,20]. The mid-cervical contusion model directly damages the phrenic motor neuron pool, resulting in loss of motoneurons [17,21,22,23]. In contrast, the C2 hemilesion model does not directly damage phrenic motoneurons but transiently paralyzes the ipsilateral diaphragm [24]. Despite differences in the spinal pathophysiology between these two injury models, both indicate that the resultant diaphragm atrophy is largely transient. Thus, the rapid reductions in myofiber size and force production tend to normalize over the weeks to months post-injury [5,13]. However, modest diaphragm fiber atrophy and contractile dysfunction can remain six weeks after injury [17,19]. Our results obtained ten days following lateral contusion injury at C3 reveal atrophy of both type I and type IIb/x diaphragm muscle fibers and significant reductions in the diaphragm force–frequency response. Importantly, daily HBO treatment improved diaphragm muscle CSA and specific force production. This is the first evidence demonstrating the therapeutic potential for HBO to promote diaphragm recovery following SCI.

### 3.2. HBO Enhances Diaphragm Oxidative Capacity and Prevents Mitochondrial ROS Emission

Oxidative stress is recognized as part of the secondary phase of SCI and occurs as a direct effect of primary spinal trauma [4]. To date, several reports confirm that disruption of phrenic output to the diaphragm augments markers of oxidative stress and ROS production [5,25], and it is hypothesized that mitochondrial dysfunction amplifies ROS release resulting in protein ubiquitination and degradation [26]. In limb skeletal muscle, both thoracic contusion injury and L1 spinal lesion result in impaired oxidative capacity and reduced mitochondrial enzyme activity and metabolism [27,28,29]. In the diaphragm, an acute C2 SCI reduces the mitochondrial respiratory control ratio and increases hydrogen peroxide emission [5]. Concurrent with these reports, our data establish that mitochondrial respiration is reduced and ROS production is elevated in the diaphragm ten days after a lateral spinal cord contusion injury at C3/4. This finding is consistent with the concept that oxidative stress is a fundamental component mediating the atrophic response in skeletal muscle [6].

Strategies designed to alleviate oxidative stress may offer effective therapeutics to prevent diaphragm weakness and support adequate pulmonary ventilation in SCI patients [4,5]. Therapies with the potential to alter skeletal muscle redox status and improve skeletal muscle morphology and function include antioxidant supplementation, exercise, neuromuscular electrical stimulation and HBO [5,10,30,31,32]. In this regard, HBO therapy has been shown to upregulate antioxidant gene expression by inducing low levels of ROS, which coincides with protection against oxidative insults [12,33,34]. Contractile dysfunction following myotoxic insult was ameliorated in the soleus muscle of injured rats that underwent daily HBO treatment at 3 ATA [9,10], and the application of HBO therapy to athletes with muscle contusion injuries accelerated recovery of muscle strength [35]. Similar to these results, HBO stimulated a significant increase in the expression of the endogenous antioxidant enzymes SOD1, SOD2 and GPX1 compared to uninjured rats. As a result, mitochondrial function was preserved and pathological mitochondrial ROS emission was stabilized in SCI rats that received HBO. Thus, HBO therapy may be an effective adjunctive treatment to preserve diaphragm muscle fiber size and contractile function acutely following cervical SCI.

### 3.3. Atrogene Expression Is Reduced in the Diaphragm Following SCI and HBO Treatment

Accelerated activity of the ubiquitin proteasome pathway is a key component of skeletal muscle atrophy, and diaphragm wasting within the first day of cervical SCI is accompanied by the upregulation of the E3 ubiquitin ligases MuRF1 and Atrogin-1/MaFbx [5,13]. In addition, MuRF1 and Atrogin-1/MaFbx gene expression is also increased in the vastus lateralis muscle of SCI patients at two- and five-days post-injury [36]. Bimodal upregulation of MuRF1 and Atrogin-1/MaFbx is also seen in gastrocnemius muscle in denervation and spinal isolation models of muscle atrophy where periods of rapid early wasting reveal greater fold increases compared to later timepoints [37]. Protein degradation is known to be tightly regulated by the transcription of these atrogenes, and knockdown of MuRF1 or Atrogin-1/MaFbx in skeletal muscle of denervated animals results in maintenance of muscle mass [38,39]. Our results confirm these findings and emphasize a requisite role for the ubiquitin proteasome pathway in mediating diaphragm proteolysis following cervical contusion injury. Our data also uncover a novel relationship between HBO exposure and atrogene expression. Specifically, increased ROS production can activate redox-sensitive transcriptions factors to induce the expression of MuRF1 and Atrogin-1/MaFbx [40,41]. Therefore, HBO therapy may prevent increased atrogene transcription by preserving diaphragm redox balance and reducing proteolysis via the ubiquitin proteasome pathway.

## 4. Materials and Methods

### 4.1. Animals

Adult male Sprague-Dawley rats were obtained from Harlan Scientific and housed at the McKnight Brain Institute Animal Care Facility at the University of Florida (UF). All experimental procedures were approved by the Institutional Animal Care and Use Committee at UF (IACUC protocol #201807438; 13 July 2018) and performed in accordance with NIH guidelines. Animals were separated into three groups *n* = 12/group: (1) non-injured, room air exposure (CON); (2) lateral-cervical spinal cord contusion, room air exposure (SCI); and (3) lateral-cervical spinal cord contusion, HBO therapy (SCI + HBOT).

#### 4.1.1. Lateral-Cervical Spinal Cord Contusion

Animals were anesthetized with inhaled isoflurane, and upon reaching a surgical plan of anesthesia an incision was made from approximately the second to the fifth cervical segment (C2–C5). Following laminectomy at the C3–C4 level, lateral-cervical spinal contusion was made using the Infinite Horizon pneumatic impactor (Precision Systems & Instrumentation, Lexington, KY, USA) as described [42]. All dependent measurements were performed on the hemi-diaphragm ipsilateral to the contusion.

#### 4.1.2. HBO Treatment

Animals were exposed to HBO for one hour per day for 10 days, initiated on the day of SCI. A custom 40 L chamber was flushed with 100% O_2_ (i.e., 1 ATA O_2_) and then pressurized to 3 ATA O_2_. During HBO exposure, the chamber was continuously flushed with gas (4 L/min) to prevent CO_2_ buildup. Twenty-four hours following the last HBO or room air exposure, animals were anesthetized with sodium pentobarbital, and the diaphragm muscle was removed.

### 4.2. Functional Analysis

#### Diaphragm Contractile function

Upon sacrifice, a diaphragm muscle strip was immediately dissected from the mid-costal region of the diaphragm. The strip was suspended vertically with one end connected to an isotonic force transducer (Aurora Scientific, Ontario, Canada) within a jacketed tissue bath. Diaphragm contractile properties were measured as described [43].

### 4.3. Histological Analysis

#### Diaphragm CSA

Immunohistochemistry was performed on a strip of mid-costal diaphragm muscle for fiber CSA analysis. Diaphragm muscle cross-sections (10 μm) were cut using a cryostat (HM 550 Cryostat, Thermo Fisher Scientific, Waltham, MA, USA) and stained for dystrophin (RB9027R7) (Thermo Fisher Scientific), myosin heavy chain Type I (A4.840) (Developmental Studies Hybridoma Bank (DSHB), Iowa City, IA, USA) and Myosin Heavy Chain Type IIa (SC-71) (DSHB). CSA was analyzed with Scion Image software (NIH, Bethesda, MD, USA).

### 4.4. Biochemical Analysis

#### 4.4.1. Permeabilized Muscle Fibers

Diaphragm muscle fiber bundles were gently separated in ice-cold buffer X (60 mM K-Mes, 35 mM KCl, 7.23 mM K_2_EGTA, 2.77 mM CaK_2_EGTA, 20 mM imidazole, 0.5 mM dithiothreitol, 20 mM taurine, 5.7 mM ATP, 15 mM PCr and 6.56 mM MgCl_2_, pH 7.1) and permeabilized in buffer X containing 75 μg/mL saponin.

#### 4.4.2. Mitochondrial Respiration

Mitochondrial oxygen consumption was measured using a respiration chamber (Hansatech Instruments, Norfolk, UK). Permeabilized muscle fibers were incubated in 1 mL of respiration buffer (100 mM KCl, 50 mM Mops, 20 mM glucose, 10 mM K_2_PO_4_, 10 mM MgCl_2_, 1 mM EGTA and 0.2% BSA, pH 7.0) at 37 °C. State 3 and State 4 respiration were assessed using complex I substrates (2 mM pyruvate and 2 mM malate) in the presence of 0.25 mM ADP as described [44]. State 3 respiration was divided by state 4 respiration to determine RCR.

#### 4.4.3. ROS Production

Diaphragm mitochondrial hydrogen peroxide production from permeabilized muscle fibers was measured continuously using a Fluorolog-3 spectrofluorometer (HORIBA, Kyoto, Japan). Measurements were performed at 37 °C using the Amplex UltraRed (Molecular Probes, Eugene, OR, USA) (10 µM)/horseradish peroxidase (HRP) (1 U/mL) system as described [45].

#### 4.4.4. Western Blotting

Diaphragm muscle was homogenized 1:10 (*w*/*v*) in 5 mM Tris (pH 7.5) and 5 mM EDTA (pH 8.0) with a protease inhibitor cocktail (Sigma-Aldrich, St. Louis, MO, USA) and centrifuged at 1500 g for 10 min at 4 °C. Protein concentration of the resultant supernatant (cytosolic fraction) was assessed by Bradford (Sigma-Aldrich). Proteins were separated via 4–20% gradient polyacrylamide gels containing 0.1% SDS and transferred to nitrocellulose membranes. Membranes were blocked for 2 h at room temperature in PBS solution containing 5% non-fat dry milk and incubated overnight at 4 °C with primary antibodies directed against SOD1 (sc-11407) (Santa Cruz Biotechnology, Dallas, TX, USA), SOD2 (sc-30080) (Santa Cruz), catalase (ab52477) (Abcam, Cambridge, MA, USA), GPXI (ab22604) (Abcam), PRXIII (sc-130336) (Santa Cruz) and α-tubulin (12G10) (DSHB). Appropriate HRP-linked secondary antibodies (anti-rabbit IgG #7074; anti-mouse IgG #7076) (Cell Signaling Technology, Danvers, MA, USA) were diluted in PBS solution containing 0.5% Tween and 5% non-fat milk. Images were acquired via chemiluminescence using Pierce ECL2 substrate (Thermo Fisher Scientific) and the G:Box imaging system (Syngene, Frederick, MD, USA) and were analyzed using ImageJ software (NIH).

#### 4.4.5. RNA Isolation and cDNA Synthesis

mRNA from diaphragm tissue was isolated using Trizol reagent (Thermo Fisher Scientific) following the manufacturer’s instructions. Total RNA content (µg/mg muscle) was evaluated by spectrophotometry. Subsequently, 5 µg RNA was reverse transcribed with the Superscript III First-Strand Synthesis System for RT-PCR (Thermo Fisher Scientific).

#### 4.4.6. Real-Time Polymerase Chain Reaction

One µl of cDNA was added to a 24 μL PCR reaction for RT-PCR using Taqman chemistry and the StepOnePlus RT-PCR system (Applied Biosystems, Foster City, CA, USA). Relative quantification of gene expression was performed using the comparative computed tomography method. SOD1 (Rn00566938_m1), SOD2 (Rn00690588_g1), catalase (Rn00560930_m1), GPX1 (Rn00577994_g1), PRXIII (Rn00574785_m1), IL-1β (Rn99999009_m1), IL-6 (Rn99999011_m1), TNFα (Rn99999017_m1), TWEAK (Rn01461586_g1), Atrogin-1/MaFbx (Rn00591730_m1) and MuRF1 (Rn00590197_m1) mRNA transcripts were assayed using predesigned rat primer and probe sequences commercially available from Thermo Fisher Scientific. β-Glucuronidase (Rn00566655_m1), a lysosomal glycoside hydrolase, was chosen as the reference gene on the basis of previous work showing unchanged expression with our experimental manipulations [5].

### 4.5. Statistical Analysis

Data are presented as mean ± standard error of the mean (SEM). Comparisons between groups for each dependent variable were made by one-way analysis of variance (ANOVA), and when appropriate, Tukey’s honestly significant difference (HSD) tests were performed post-hoc. Significance was established at *p* < 0.05.

## 5. Conclusions

The results from this study reveal for the first time that HBO delivered in the acute phase following cervical spinal contusion is sufficient to prevent reductions to diaphragm muscle fiber size and contractility. This is important because diaphragm muscle function is essential not only for breathing after SCI, but also associated behaviors such as coughing and sighing. The inability to produce large force behaviors required for coughing, for example, contributes to lung infections, morbidity and mortality after SCI. Preservation of diaphragm function could mitigate respiratory-related problems. Another consideration is the high incidence of mechanical ventilation in patients with severe cervical SCI. Once ventilator support is initiated, ventilator-induced diaphragm dysfunction can negatively affect diaphragm contractility and weaning outcomes [5,14]. In this regard, the upregulation of antioxidant mechanisms by acute HBO therapy could be beneficial. Lastly, the current findings complement initial indications that HBO therapy can mitigate neuropathology and improve motor function after SCI recovery [46,47,48]. We conclude that HBO has the potential to enhance respiratory recovery following SCI and merits further study to determine optimal timing and dosing strategies.
